# High-level diterpene production by transient expression in *Nicotiana benthamiana*

**DOI:** 10.1186/1746-4811-9-46

**Published:** 2013-12-12

**Authors:** Kathleen Brückner, Alain Tissier

**Affiliations:** 1 Department of Cell and Metabolic Biology, Leibniz Institute of Plant Biochemistry, Weinberg 3, 06120 Halle-Saale, Germany

**Keywords:** Diterpene synthase, Agrobacterium, Transient protein expression, *Nicotiana benthamiana*

## Abstract

**Background:**

Characterization of plant terpene synthases is typically done by production of recombinant enzymes in *Escherichia coli*. This is often difficult due to solubility and codon usage issues. Furthermore, plant terpene synthases which are targeted to the plastids, such as diterpene synthases, have to be shortened in a more or less empirical approach to improve expression. We report here an optimized Agrobacterium-mediated transient expression assay in *Nicotiana benthamiana* for plant diterpene synthase expression and product analysis.

**Results:**

Agrobacterium-mediated transient expression of plant diterpene synthases in *N. benthamiana* led to the accumulation of diterpenes within 3 days of infiltration and with a maximum at 5 days. Over 50% of the products were exported onto the leaf surface, thus considerably facilitating the analysis by reducing the complexity of the extracts. The robustness of the method was tested by expressing three different plant enzymes, cembratrien-ol synthase from *Nicotiana sylvestris*, casbene synthase from *Ricinus communis* and levopimaradiene synthase from *Gingko biloba*. Furthermore, co-expression of a 1-deoxy-D-xylulose-5-phosphate synthase from tomato and a geranylgeranyl diphosphate synthase from tobacco led to a 3.5-fold increase in the amount of cembratrien-ol produced, with maximum yields reaching 2500 ng/cm^2^.

**Conclusion:**

With this optimized method for diterpene synthase expression and product analysis, a single infiltrated leaf of *N. benthamiana* would be sufficient to produce quantities required for the structure elucidation of unknown diterpenes. The method will also be of general use for gene function discovery, pathway reconstitution and metabolic engineering of diterpenoid biosynthesis in plants.

## Background

Terpenoids constitute a highly diverse group of compounds which are found in a wide variety of plants and microbial species as well as in animals. Their structural complexity is based on two comparatively simple basic C5 building blocks, isopentenyl diphosphate (IPP) and dimethylallyl diphosphate (DMAPP). Sequential head-to-tail coupling of IPP to allylic isoprenyl diphosphates, starting with DMAPP, gives rise to isoprenyl moieties of increasing lengths, namely geranyl diphosphate (GPP, C_10_), farnesyl diphosphate (FPP, C_15_) and geranylgeranyl diphosphate (GGPP, C_20_). These isoprenyl diphosphates are the substrates of a large class of enzymes, the terpene synthases (TPS), which, through their cyclase activity, are the key contributors to the structural diversity of terpenes. Accordingly, terpenes are classified according to the number of isoprenyl units they contain in monoterpenes (C_10_), sesquiterpenes (C_15_) and diterpenes (C_20_). Many terpenoid compounds are of great importance for human nutrition, fragrance industry and medicine. Several plant diterpenoids in particular are produced commercially, such as the anti-cancer compound Taxol, the labdanoid sclareol as a precursor for fragrance ingredient, or carnosic acid and carnosol as anti-oxidants for cosmetic and food applications [1-3]. To increase the yield of these diterpenoids or to gain better access to the diversity of diterpenoids by metabolic engineering in microbial or plant hosts there is an increasing interest in elucidating biosynthesis pathway genes.

*Escherichia coli* has been typically employed for the biochemical characterization of terpene synthases because of the ease of manipulation and of the access to a wide range of expression hosts and vectors. A large number of plant mono- and sesquiterpene synthases have been successfully expressed in *E. coli* and isolated for *in vitro* enzyme assays [4]. Heterologous protein expression using microbial hosts has also been widely applied for the screening of diterpene synthase function. One of the best studied example is taxadiene synthase from *Taxus sp.* which synthesizes taxa-4(5),11(12)-diene, a key intermediate for the biosynthesis of the anti-cancer drug paclitaxel (Taxol) [5,6]. By using recombinant *E. coli* strains, some further diterpene synthases have been identified, such as *z*-abienol synthase from *Nicotiana tabacum* and bifunctional levopimaradiene/abietadiene synthases from *Pinus sp.*, respectively [7,8].

However, using microbial hosts presents some drawbacks for overexpression of plant terpene synthases. In particular, the proteins may not fold properly and form aggregates that are stored in inclusion bodies as reported for casbene cyclase production in *E. coli*[9]. Misfolding could be caused by the presence of N-terminal signal peptides which are non-functional in microbial hosts. Foreign protein production in yeast and *E. coli* can also be accompanied by difficulties in plasmid stability and codon usage. Furthermore, toxicity of the target gene sequence or of the catalytic activity of the protein in combination with already low expression levels could prevent the production of functional recombinant protein. Nonetheless, there have been successful attempts at producing diterpenes *in vivo* in *E. coli* or yeast hosts [10,11]. In these cases however, the production of terpenoids can be limited by intracellular levels of GGPP or toxicity of the diterpenoids to the host. Most prokaryotes including *E. coli* and plant plastids synthesize IPP and DMAPP through the 2-C-methyl-D-erythritol-4-phosphate (MEP) pathway while most eukaryotes synthesize IPP via the mevalonate (MEV) pathway. Increasing the flux by single-enzyme introduction or exogenous precursor supply may improve productivity [12]. On the other hand, the introduction of a MEV pathway in *E. coli* was reported to induce growth inhibition triggered by the accumulation of HMG-CoA [13], but successive improvements led to high production levels of amorphadiene, the precursor of the anti-malaria compound, artemisinin [14,15]. Yeast is an attractive alternative to *E. coli*, in particular because the expression of ER-bound plant cytochrome P450 oxygenases, which are frequently involved in the biosynthesis pathways of plant terpenoids, is more easily implemented in yeast than in *E. coli* due to the absence of ER in bacteria. Thus a number of reports describe the use of yeast to produce plant diterpenoids [16-22]. So far, the production yields of diterpenes in yeast rarely exceed several hundred mg/mL, thus far below the rates achieved for the production of artemisinic acid in yeast (25 g/L) [23]. The reasons for this are not clear but could have to do with general toxicity of diterpenes towards yeast. Furthermore, since plant diterpene synthases are usually targeted to the plastids, optimization of expression in yeast is achieved through empirical shortening of the target peptide.

*Nicotiana benthamiana*, which is now widely used for transient expression assays in plants, is an alternative host which may circumvent the need for optimization required with microbial hosts. Since plants have both functional MEV and MEP pathways, extensive engineering of these pathways can be avoided to detect the products of terpene synthases. Furthermore, expression *in planta* allows investigating issues such as sub-cellular localization. *N. benthamiana* has originally been used as host in plant virology, and is now widely employed for transient protein expression [24]. The method of agro-infiltration is highly efficient in *N. benthamiana* and permits proteins of interest to be produced transiently in plant cells [25,26]. Recently, *N. benthamiana* was used successfully for the production of plant terpene synthases, namely amorpha-4,11-diene synthase and *epi*-cedrol synthase from *Artemisia annua*[27,28]. These investigations revealed that *N. benthamiana* can be used to produce recombinant biosynthetic enzymes in relatively high yields of 90 and 96 mg protein per kg fresh weight of infiltrated leaves, respectively. Expression in *N. benthamiana* was also used successfully for examining the biochemical function of plant mono-TPS linalool synthase ApLS1 from kiwifruit [29], and of several sesqui-TPS enzymes [27,30] by directly detecting the products, thus bypassing the need of *in vitro* assays. This approach was also effective for the expression of triterpene biosynthesis enzymes including an acyl-transferase and a multifunctional cytochrome P450 [31,32].

These considerations led us to adapt the *N. benthamiana* transient expression system for the functional analysis of diterpene synthases. A simple and efficient method for *in planta* expression of various plant diterpene synthases including the rapid detection of the corresponding products by GC-MS is described.

## Results

### High-level diterpene yield in exudate of agro-infiltrated *N. benthamiana* leaves

To test *N. benthamiana* as a transient expression system for the production of plant diterpenes, we chose the cembratrien-ol (CBT-ol) synthase encoded by the trichome-specific *NsCBTS2a* gene from *Nicotiana sylvestris*[33]. CBTS enzymes from tobacco are class I terpene synthases which directly cyclize GGPP to a mixture of α- and β-CBT-ol.

**Figure 1:**
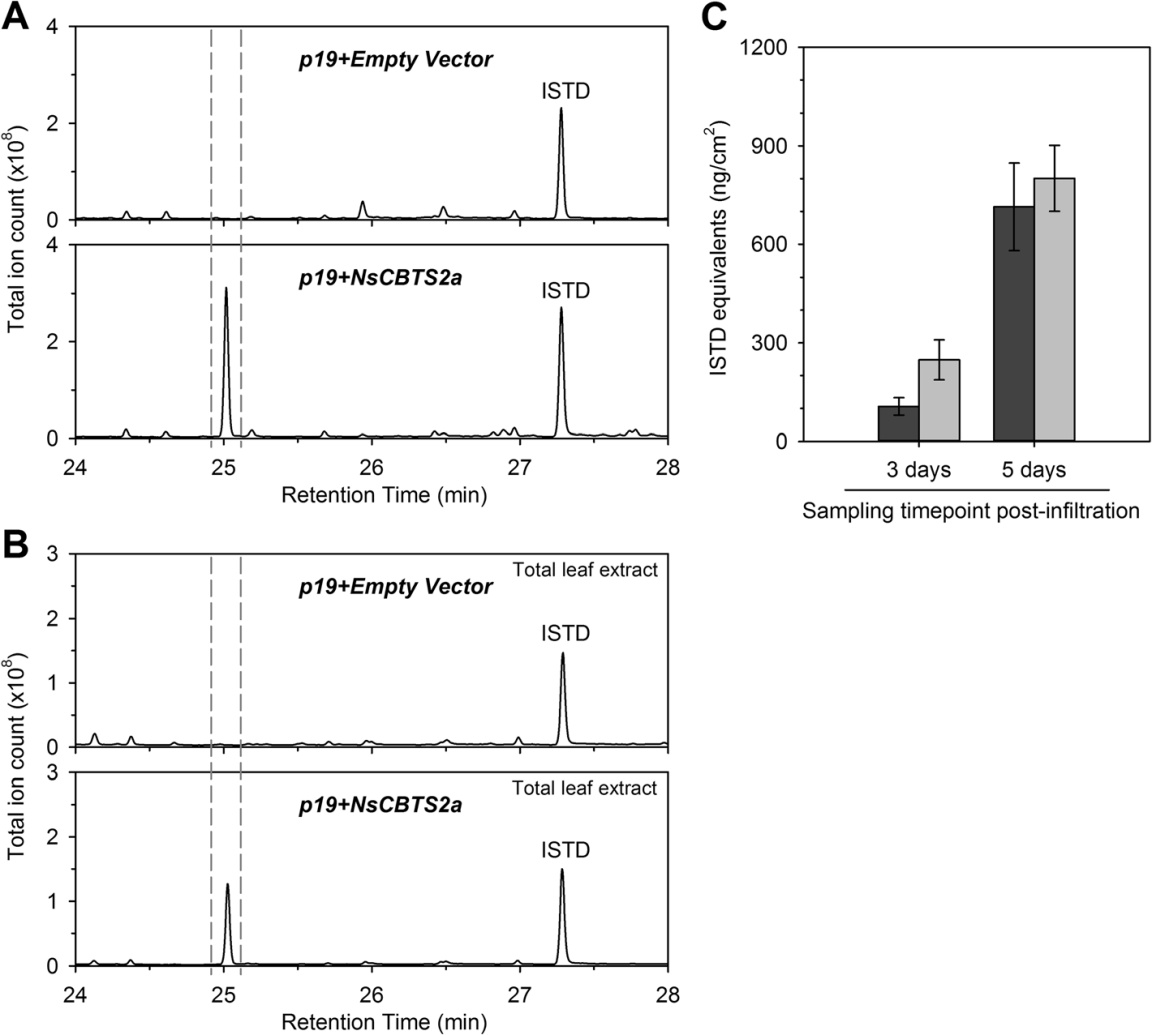
**Production of CBT-ol. (A)** Total ion chromatograms of hexane washes from agro-infiltrated *N. benthamiana* leaves co-expressing *p19* and *NsCBTS2a* or the empty T-DNA vector (pL1F-1) as a control. The CBT-ol peak is framed by vertical dashed lines. The α- and β-stereoisomers of CBT-ol could not be separated under standard GC-MS conditions. **(B)** Total ion chromatograms of total extracts from agro-infiltrated *N. benthamiana* leaves. The extracts were purified over SPE column prior to GC-MS analysis. **(C)** CBT-ol content in agro-infiltrated leaves harvested three and five days after transformation of *N. benthamiana* plants with *NsCBTS2a* and *p19*. Hexane washes from the surface of treated leaves (black bars) and hexane extracts from the rest of the leaf material (total extracts, grey bars) were analyzed by GC-MS. No CBT-ol was detected in leaves transformed with the empty T-DNA vector (see Additional file 3). Samples were taken from two *N. benthamiana* plants, in each case from two infiltrated leaves. Mean CBT-ol values ± standard error of the mean (SEM) are presented (n = 8). There is a statistically significant difference (*P* < 0.001, two-way-ANOVA) in the mean CBT-ol values regarding the timepoint of sampling post-infiltration. ISTD – internal standard sclareol.

*Agrobacterium* strains containing T-DNA constructs with the full length *CBTS2a* coding sequence under the control of a Cauliflower Mosaic Virus (CaMV) 35S promoter were used to infiltrate *N. benthamiana* leaves. CBTS2a was co-expressed with viral-encoded protein p19 that has been shown to suppress post-transcriptional gene silencing in host cells thereby allowing high levels of transient expression [26]. Five days after infiltration, the surface of leaf discs from the infiltrated parts was extracted with hexane and analyzed by GC-MS. The CBT-ol peak was clearly detectable (Figure 1A) and identified by comparison of mass spectra (see Additional file 1) with published references and extracts of *N. tabacum* cultivars which produce significant amounts of CBT-ol [8,33]. A time-course analysis confirmed that CBT-ol accumulates to significant amounts 3 days post-infiltration and strongly increases 5 days post-infiltration (Figure 1C).

To see what portion of CBT-ol produced during transient expression remains in the leaf, extracts from leaves which had been washed with hexane were prepared and the CBT-ol content was estimated by GC-MS (Figure 1B, see Additional file 2 and Additional file 3). In comparison to the CBT-ol yield in hexane washes, a slightly larger portion of CBT-ol remained inside the leaves three days post-infiltration (Figure 1C). Five days after infiltration however, comparable amounts were detected from the internal and secreted fractions. As few as six leaf discs (diameter 9 mm) from infiltrated areas of *N. benthamiana* were required for the detection of CBT-ol from leaf washes.

### Efficient expression of various plant diterpene synthases

**Figure 2:**
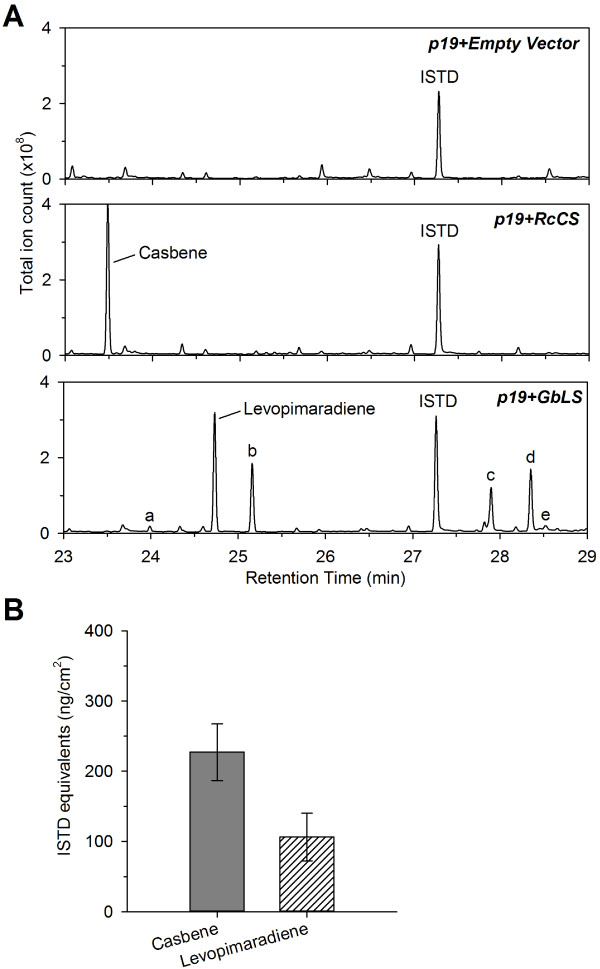
**Production of casbene and of levopimaradiene. (A)** Total ion chromatograms of hexane washes from *N. benthamiana* leaves agro-infiltrated with *p19* and either the empty T-DNA vector (control) or *RcCS* or *GbLS*. Peaks for casbene, levopimaradiene and other novel products (a – e) are indicated. **(B)** Casbene and levopimaradiene content in hexane washes from leaves agro-infiltrated with *p19* and *RcCS* or *GbLS*, respectively. Samples were taken five days post-infiltration from two individual *N. benthamiana* plants, each of them having two completely infiltrated leaves. Mean casbene and levopimaradiene values ± SEM are presented (n = 16, two independent experiments). Sclareol was used as internal standard (ISTD) for quantification.

To test the robustness of our assay, other diterpene synthases were expressed under the same conditions. *Ricinus communis* casbene cyclase (RcCS) was first tested and co-expressed with p19. Like NsCBTS2a, RcCS is a class I terpene synthase. It catalyzes the cyclization of GGPP to casbene, a diterpene phytoalexin with antifungal activity in germinating seedlings of castor bean [34]. Casbene was readily detected in hexane washes from treated tissue five days post-infiltration, as indicated by GC-MS analysis (Figure 2A). Lacking commercially available standards, confirmation of casbene identity was based on the comparison of experimental mass spectra (see Additional file 1) with published spectra for this compound [10]. Simple leaf dipping was sufficient for the purification of up to 227 ng/cm^2^ casbene (in sclareol equivalent) from treated tissue (Figure 2B).

Levopimaradiene synthase from *Ginkgo biloba* (GbLS) was expressed under the same conditions. This bifunctional diterpene synthase catalyzes a multi-step reaction in which the precursor of the pharmaceutically important ginkgolides, levopimaradiene, is formed [35]. Upon expression of GbLS in *N. benthamiana*, levopimaradiene was produced and identified (Figure 2A, see Additional file 1) based on published references [35,36]. Noticeably, the yield of levopimaradiene in surface extracts of infiltrated leaves was lower compared to those observed for CBT-ol and casbene upon transient expression of the corresponding enzymes, and rarely exceeded 100 ng/cm^2^ five days post-infiltration (Figure 2B). The expression of LS in *N. benthamiana* further resulted in the formation of other diterpene products (Figure 2A, peaks a and b). One of those compounds was identified as abietatriene (peak b, see Additional file 4) which has been reported previously as a dehydrogenated derivative of levopimaradiene and found in roots of *Gingko biloba* seedlings [37]. The identity of peak a could not be defined. Its mass spectrum (see Additional file 4) looked highly similar to that of sandaracopimaradiene which was detected in culture media of an engineered *E. coli* strain expressing LS [38], and to that of a pimaradiene-like hydrocarbon which was found in roots of *Ginkgo biloba* seedlings in trace amounts [35,37].

Furthermore, the production of levopimaradiene was invariably accompanied by the conspicuous appearance of novel peaks eluting later in the chromatogram (Figure 2A, peaks c-e). The extracted mass spectra of these peaks (see Additional file 4) showed characteristic signals at 288 m/z (peak c) and 286 m/z (peaks d and e). Furthermore, the mass spectrum of peak c is strongly reminiscent of that of levopimaradiene, and that of peaks d and e to that of abietatriene. Although the identity of the compounds could not be established, these observations suggests that they correspond to mono-hydroxylated derivatives of levopimaradiene (peak c) and abietatriene (peaks d and e), respectively. They likely originate from the activity of endogenous hydroxylases from *N. benthamiana*.

### Optimization of diterpene production

A common strategy to increase production of high-value compounds in heterologous expression systems such as *E. coli* or yeast focused on overexpression of pathway enzymes and improving enzyme activities by codon optimization [12]. Concerning the MEP pathway, it could be shown for example in *E. coli* that the first step of the pathway, 1-deoxy-D-xylulose-5-phosphate synthase (DXS), constitutes a bottleneck which can be overcome by overexpression [39,40]. A recent example of DXS over-expression in *Arabidopsis* resulted in a 6-fold increase of taxadiene production compared to the transgenic plant line expressing only taxadiene synthase [41]. Similar effects are reported for geranylgeranyl diphosphate synthase (GGPPS) which uses IPP and DMAPP to synthesize geranylgeranyl diphosphate as the main precursor for a large set of different plastidic isoprenoids. For example, overexpression of GGPPS has been successfully used to boost miltiradiene production in *Salvia miltiorrhiza* hairy root cultures and in *Saccharomyces cerevisiae* cultures [11,42]. To investigate whether over-expression of the initial step of the MEP pathway or of GGPPS impact CBT-ol production, DXS2 from *Solanum lycopersicum*[43] and GGPPS2 from *Nicotiana tabacum*[44] were cloned and expressed alone or in combination with CBTS2a in leaves of *N. benthamiana*. For direct comparison of CBT-ol yield between different assays, the specific agrobacteria mixtures were infiltrated into separate parts on the same leaf. The hexane dipping procedure was used for extraction of CBT-ol from treated tissue. As controls, *in planta* expression of the individual enzymes, DXS2, p19 and GGPPS2, was performed and as expected did not induce any production of CBT-ol (see Additional file 5).

**Figure 3:**
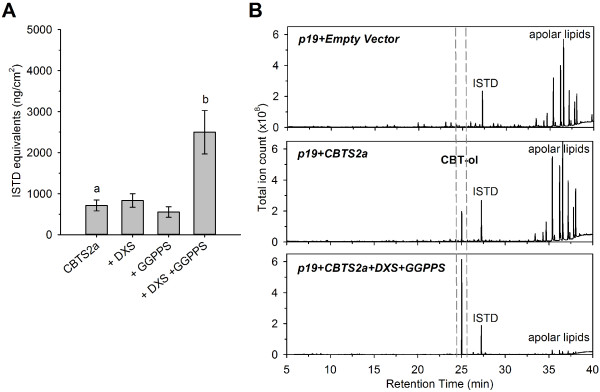
**Increasing expression levels of isoprenoid precursor pathway genes DXS and GGPPS lead to increased CBT-ol production in agro-infiltrated *N. benthamiana *leaves. (A)** CBT-ol content in hexane washes from *N. benthamiana* leaves co-expressing *p19* and *NsCBTS2a* with *SlDXS2* and/or *NtGGPPS2* in different combinations. The appropriate *Agrobacterium* cultures were infiltrated into separate parts of the same *N. benthamiana* leaf and in two leaves per individual plants. Leaves were harvested five days post-infiltration. Mean CBT-ol values ± SEM are presented (n = 8). Groups indicated by different letters differ significantly from each other regarding their CBT-ol values (*P* < 0.05, Student’s *t*-test and one-way ANOVA). ISTD – internal standard sclareol; **(B)** Full total ion chromatograms demonstrating high CBT-ol peak (dashed lines) when *NsCBTS2a* was co-expressed with both *SlDXS2* and *NtGGPPS2*. No CBT-ol was detected in leaves agro-infiltrated with the empty T-DNA vector as a control.

GC-MS analysis of extracts revealed that co-expression of CBTS2a with DXS2 or GGPPS2 alone does not alter CBT-ol production significantly compared to leaves solely infiltrated with CBTS2a (Figure 3A). In contrast, co-expression of DXS2 and GGPPS2 with CBTS2a resulted in a significant 3.5-fold increase in the production of CBT-ol (Figure 3A). Under these conditions, the CBT-ol peak was the highest from the whole chromatogram, exceeding the levels of the cuticular lipids which are typically dominant in such extracts (Figure 3B).

## Discussion

Our results demonstrate that the *N. benthamiana* transient expression system described here is a rapid and easily applicable method for testing the functionality of various plant diterpene synthases. NsCBTS2a, RcCS and GbLS were successfully expressed in leaves of *N. benthamiana,* as documented by GC-MS analysis of plant extracts showing the expected product specificity of the recombinant enzymes. Thus, class I as well as bifunctional plant diterpene synthases seem to work equally well in this system. It is intriguing that only GbLS gives rise to significant oxidation products, but it is unlikely that this has something to do with its bifunctionality (see below). Further, it was possible to extract a majority of the reaction products from the surface of infiltrated leaves by organic solvent dipping. The exuded products may derive from agro-treated *Nicotiana benthamiana* trichomes and epidermal cells whose surface is extracted upon short exposure to hexane. Two types of trichomes were reported in *N. benthamiana*: long stalked glandular hairs with swollen bases and smaller capitate trichomes with thin stalks and single or multiple-celled secretory heads and were demonstrated to be active in acyl sugar synthesis [45]. So far, it is not known whether *Agrobacterium* would be able to infect trichomes or whether *N. benthamiana* would secrete diterpenoid products into the hairs. However, glandular trichomes from *N. benthamiana* are not known to produce terpenoids. Furthermore, the large amounts of CBT-ol that were observed in the combined expression assays exceed that of individual cuticular components. These observations suggest that epidermal cells are the main contributors to the diterpenes that are extracted in the leaf washes. In any case, the extraction of leaf surface allows a fast and simple way to identify the products of the expressed diterpene synthases, and could replace recently used labor-extensive methods which include grinding of plant tissue followed by sequential extraction and purification steps prior to chromatography [46]. In addition, because surface extracts are much less complex than whole leaf extracts, this procedure should allow the facile purification of novel products for structure elucidation or for their use as authentic standards for quantification. It should be noted however that this method is likely not to be suitable for smaller terpenes (i.e. mono- and sesquiterpenes) which, because of their volatility, are unlikely to accumulate at the surface of *N. benthamiana* leaves. These can be detected by headspace measurements as was previously shown [30]. With the optimized yields that we obtained for CBT-ol, infiltration of as little as 30 cm^2^ (one leaf) of *N. benthamiana* would suffice to recover 100 μg of CBT-ol, a quantity which is sufficient to produce NMR spectra. Optimization of CBT-ol production was achieved by co-expressing CBTS with both the DXS2 and GGPPS2 enzymes. When synthesis was coupled to the expression of either DXS2 or GGPPS2 alone, the CBT-ol yield did not increase suggesting that the endogenous GGPPS activity is not limiting but would become so when DXS alone is over-expressed. Thus a significant increase in flux through the pathway could only be achieved when both enzymes are over-expressed. It is also possible that the increase of DXS activity could result in the accumulation of intermediates that contribute to negative feedback regulation of the pathway. Methylerythritol cyclodiphosphate (MEcPP), a MEP pathway intermediate, could be such a regulatory compound, since it was recently shown in Arabidopsis to act as a retrograde signal molecule which elicits a stress response [47]. Further analysis of the impact of MEcPP on the expression of MEP pathway genes would need to be carried out to validate this hypothesis. In addition, extensive metabolite profiling of DXP pathway intermediates together with flux analyses would be required to clarify this issue. Moreover, further optimization may be possible by over-expression of other MEP pathway genes, along with DXS and GGPPS.

Although no significant accumulation of by-products could be detected for CBT-ol and casbene, expression of GbLS was accompanied by the modification of levopimaradiene and abietatriene, most likely hydroxylation by endogenous *N. benthamiana* oxidases. To the best of our knowledge, direct modification of exogenous terpene olefins in *N. benthamiana* has not been reported yet. However, hydroxylated or carboxylated terpenoids such as artemisinic acid or germacrenoic acid were shown to undergo several types of modifications, including glycosylation and glutathione-transfer [27,30]. The fact that no modification could be detected with casbene or CBT-ol suggests that the modifications are specific to the products of GbLS. One possibility is that one of these products induces a detoxification response in *N. benthamiana* resulting in the expression of oxidases, e. g. P450 mono-oxygenases, which would then hydroxylate levopimaradiene and abietatriene. Alternatively, such oxidases could be constitutively expressed and would exhibit some degree of product specificity since casbene and CBT-ol do not seem to be modified by these activities. Interestingly, such abietane-type diterpenes, including levopimaradiene, are extensively oxidized in various plant species, particularly in the conifers and Lamiaceae, and it is therefore likely that similar processes take place in tobacco [16,48-50]. To further clarify this point, systematic testing of diverse diterpene synthases should indicate to what extent this response is specific of certain diterpenes.

## Conclusion

The characterization of plant diterpene synthases by transient expression in *N. benthamiana* with our optimized method offers a number of advantages over expression in microbial hosts (*E. coli* or yeast). First, subcellular targeting is not an issue any more since the processing of organellar targeting signals is highly conserved in plants. Thus, full length coding sequences can be used directly for expression without concerns about removing a signal peptide whose length still has to be determined empirically. Second, solubility and functionality of the expressed protein is often an issue when expressing plant enzymes in *E. coli* or yeast, particularly with terpene synthases. Here, although the diterpene synthases tested come from highly divergent plant species, they could all be functionally expressed without optimization of the coding sequence. Finally, in comparison to transgenic plants, the transient system provides a rapid method, reduced costs of production and scalability thanks to the large amounts of tissue that can be infiltrated. Co-expression with SlDXS2 and NtGGPPS2 ensures that sufficient quantity of the product for structure elucidation can be recovered with a relatively small sample size, i.e. one leaf. Beyond the characterization of diterpene synthase products, our optimized method should be of general use for the reconstitution of diterpenoid biosynthetic pathways and combinatorial biosynthesis.

## Methods

### Plant material

*Nicotiana benthamiana* seeds were germinated directly in soil and grown in climate-controlled chambers with a 16-h day/8-h night photoperiod under an illumination of 90 μmol/s/m^2^ generated by MT250DL metal halide lamps. Temperatures in the growth chamber were set to 20°C during the day and to 18°C in the night. Humidity was adjusted to 60%.

### Oligonucleotide primers

All oligonucleotide primers which were used for the isolation of cDNA for cloning and in PCR reactions are listed in Additional file 6. The cloning primers were designed by hand with artificial *BpiI* restriction sites at the 5’ and 3’ end necessary for Golden Gate cloning [51] procedures.

### T-DNA constructs for in planta expression

Recombinant plasmids were generated according to the Golden Gate cloning method [51,52]. Full length coding sequences for NsCBTS2a, RcCS, GbLS, SlDXS2 and NtGGPPS2 were PCR amplified using Phusion Hot Start II High-Fidelity DNA Polymerase (Thermo Scientific, http://www.thermoscientific.com) and gene specific primers. Prior to cloning, any BpiI and BsaI restriction sites that were present within gene sequence were removed by mutating single nucleotides of enzyme recognition pattern and dividing the gene into sub-fragments devoid of these sites according to [51]. Entry modules were generated by cloning the sub-fragments into vector pGEM-T-easy (Promega, http://www.promega.com) according to the manufacturer’s instructions or in pUC18 according to Engler and Marillonnet [53]. The entry modules were assembled to full length gene into Golden Gate entry vector pL0-SC [54] using BpiI (Fermentas, http://www.thermoscientificbio.com/fermentas) and Promega T4-HC-Ligase, and the inserted fragment was verified by sequencing. Finally, the target genes in pL0-SC were transferred into T-DNA vector pL1F-1, and fused to CaMV 35S promoter and Nos-terminator by a single reaction using BsaI (New England Biolabs, http://www.neb.com) and T4-HC-Ligase (Promega).

### Transient expression in *Nicotiana benthamiana*

T-DNA plasmids containing diTPS gene insertion were transformed into *Agrobacterium tumefaciens* strain GV3101::pMP90 [54] by electroporation. The agrobacteria were streaked on LB agar plates containing rifampicin (25 μg/ml), gentamycin (50 μg/ml) and carbenicillin (50 μg/ml). The plates were incubated for 2-3 days at 28°C and colonies were tested for insertion by PCR using DreamTaq DNA Polymerase (Thermo Scientific) and gene specific primers. Transformed single colony was used to inoculate a 5 ml culture of LB medium plus appropriate antibiotics and cells were grown for 24 hours at 28°C and 180 rpm. This pre-culture was used to inoculate a 50 ml culture and grown for another 16-20 hours at 28°C. The OD (600 nm) was adjusted to 0.5 in a total volume of 20 ml. Agrobacteria were harvested by centrifugation at 1800 × *g* and 4°C for 90 min. When several constructs were co-infiltrated, the corresponding agrobacteria batches were mixed together in equal proportion before centrifugation and so that the OD_600_ = 0.5. The cell pellet was resuspended in 0.25 volumes LB medium, 0.25 volumes sterile water and 0.5 volumes of infiltration buffer (10% w/v sucrose, 20 mM glucose, 8.6 g/l Murashige & Skoog basal salt mixture) to reach a final volume of 20 ml. 20 μM Acetosyringone were added to the agrobacteria suspension and the mixture was immediately infiltrated into leaves of 4-5 weeks old *N. benthamiana* plants by pressing a 1 ml syringe against the abaxial side of the leaf. Transformed plants were returned to climate-controlled chambers for 3-5 days until further analysis. *Agrobacterium* strain GV3101 carrying a T-DNA expressing the p19 protein was co-infiltrated to improve transgene expression by suppressing post-transcriptional gene silencing [26].

### Metabolite extraction

To isolate diterpenoid compounds produced in leaves agro-infiltrated with diTPS expression constructs, six leaf discs (9 mm in diameter) were prepared from infiltrated leaf area and extracted by dipping in 1 ml hexane for 2 min at room temperature. The extract was evaporated completely under a stream of nitrogen, and resuspended in 200 μl of hexane supplied with 20 μM of internal standard sclareol (Sigma-Aldrich, http://www.sigmaaldrich.com). The extracts were centrifuged at 16.000 × *g* for 5 min prior to their transfer into GC vials.

After having extracted the leaf surface, the leaf discs were transferred into test tubes (Biozym Scientific, http://www.biozym.com) containing five steel beads (3 mm in diameter), and quickly frozen in liquid nitrogen. Frozen leaf discs were homogenized to a fine powder using Mixer Mill MM-400 (Retsch, http://www.retsch.de) and two runs at 30 Hz for 30 sec, and followed by using FastPrep-24 instrument (MP Biomedicals, http://www.mpbio.com) for 20 sec at 4 m/s with the samples stored on dry ice. Samples were mixed with 1 ml hexane and extraction was carried out via FastPrep-24 system for 20 sec at 6 m/s, once with the samples stored on dry ice and two more times at room temperature. The extracts were clarified by centrifugation at 16.000 × *g* and 4°C for 15 min, and further purified using unmodified Chromabond-SiOH SPE columns (Macherey-Nagel, http://www.mn-net.com). The SPE column was equilibrated with one volume of hexane, followed by the loading of the sample extract. The column was washed with 1 ml hexane. Afterwards, five fractions of each 1 ml were collected using hexane/ethyl-acetate/methanol (90:10:1 by volume) as eluent. The fractions containing the product of interest (see Additional file 2) were combined and the eluate was concentrated under nitrogen flow in a 1.5 mL microcentrifuge tube, and the residue was dissolved with 400 μl hexane. Before transferring the extracts into GC vials, the samples were centrifuged at 16.000 × *g* for 2 min and mixed with internal standard sclareol giving a final concentration of 20 μM.

### GC-MS analysis of solvent extracts

For GC-MS analysis, 1 μL of the samples was injected directly into a Trace GC Ultra gas chromatograph coupled to ISQ mass spectrometer (Thermo Scientific). Separation was achieved in a 30-m × 0.32-mm diameter capillary, with a 0.25-μm film of ZB-5 ms (Phenomenex, http://www.phenomenex.com). Splitless mode was used for the injector with the inlet temperature set to 250°C. The oven was programmed to start at 50°C and a 1-min hold, after which temperature increased to 300°C at a rate of 7°C min^-1^ and further to 330°C with 20°C min^-1^ and a 5-min hold. Helium was used as carrier gas and was adjusted to a flow rate of 1 ml/min. Electron impact was recorded at 70 eV and MS data were collected from 50 to 450 *m/z* during the temperature ramp. Diterpenoids were quantified by determination of their total ion count (TIC) peak area and comparison to the peak area of the internal standard (ISTD) sclareol. The amount of target compounds is given as ISTD equivalents.

### Accession numbers

Sequence data from this article can be found in public databases under the following accession numbers: [GenBank:HM241151 (NsCBTS2a), GenBank:L32134 (RcCS), GenBank:AF331704 (GbLS), GenBank:FN424052 (SlDXS2), GenBank:GQ911584 (NtGGPPS2)].

## Competing interests

The authors declare that they have no competing interests.

## Authors’ contributions

AT conceived the study, KB and AT designed the experiments, KB carried out the experiments, KB and AT interpreted the results and wrote the manuscript. KB and AT read and approved the final manuscript. AT submitted the manuscript.

## Supplementary Material

Additional file 1 Mass spectra recorded for the main products of the diterpene synthases investigated in this study: (A), CBT-ol, measured mass spectrum and published reference mass spectra; (B), Levopimaradiene; and (C) Casbene. Click here for file

Additional file 2 **GC-MS analysis of SPE eluates from total extracts of *N. benthamiana* leaves agro-infiltrated with *p19* and *NsCBTS2a*.** Treated leaves were harvested five days post-infiltration and crude extracts from six frozen leaf discs (9 mm in diameter) were purified over SPE column using hexane/ethyl-acetate/methanol (90:10:1, v/v) as eluent. The CBT-ol peak is framed by vertical dashed lines. Click here for file

Additional file 3 **GC-MS analysis of total extracts and hexane washes of *N. benthamiana* leaves co-expressing *p19* and the empty T-DNA vector (pL1F-1) as a control.** Treated leaves were harvested five days after infiltration of the plants. A) 272 *m/z* extracted spectra of SPE eluates from total leaf extracts after purification over SPE column. B) 272 *m/z* extracted total ion chromatograms of hexane washes from three independent leaf samples. No specific peak for 272 *m/z* was detected in the extracts indicating that no CBT-ol, casbene or levopimaradiene can be found in *N. benthamiana* upon co-expression of *p19* and the empty T-DNA vector. Click here for file

Additional file 4 **GC-MS analysis of reaction products a – e which occurred in *N. benthamiana* leaves agro-infiltrated with *p19 *** **and *GbLS*.** (A) 272 *m/z* extracted ion chromatograms and full scan mass spectra for diterpene-like compound peaking at 23.97 min (peak a). (B) 270 *m/z* extraction for peak b which was identified as Abietatriene using NIST MS search software v2.0 (http://chemdata.nist.gov/) (C) 288 *m/z* extracted chromatogram and corresponding mass spectrum for product peak c eluting after 27.88 min. (D) 286 *m/z* extracted ion chromatograms and the mass spectra recorded for compounds detected at 28.33 min (peak d) and 28.49 min (peak e), respectively. Click here for file

Additional file 5 **Sections of total ion chromatograms obtained by GC-MS analysis of hexane washes from leaves infiltrated with *p19* alone or co-infiltrated with *p19* and *SlDXS2 *** **or *NtGGPPS2 *** **as controls.** The infiltrated leaves were harvested five days post-infiltration. Click here for file

Additional file 6 List of oligonucleotide primers which were used for PCR and Golden Gate cloning procedures. Click here for file
