# Supplementary figures and images for: High-level diterpene production by transient expression in Nicotiana benthamiana
Source: Plant Methods. 2013 Dec 12;9:46. doi: 10.1186/1746-4811-9-46 (PMC3878842; doi:10.1186/1746-4811-9-46)

**A**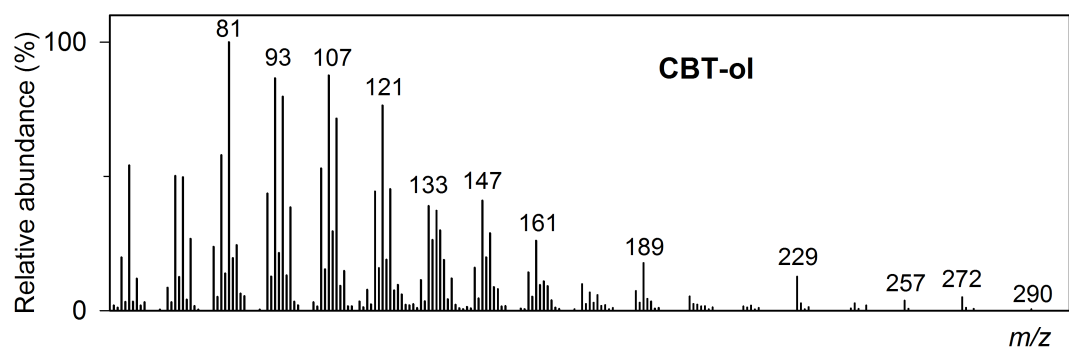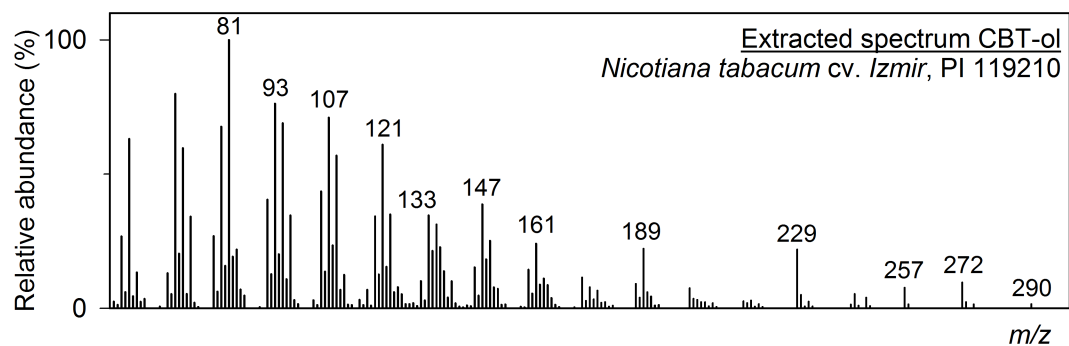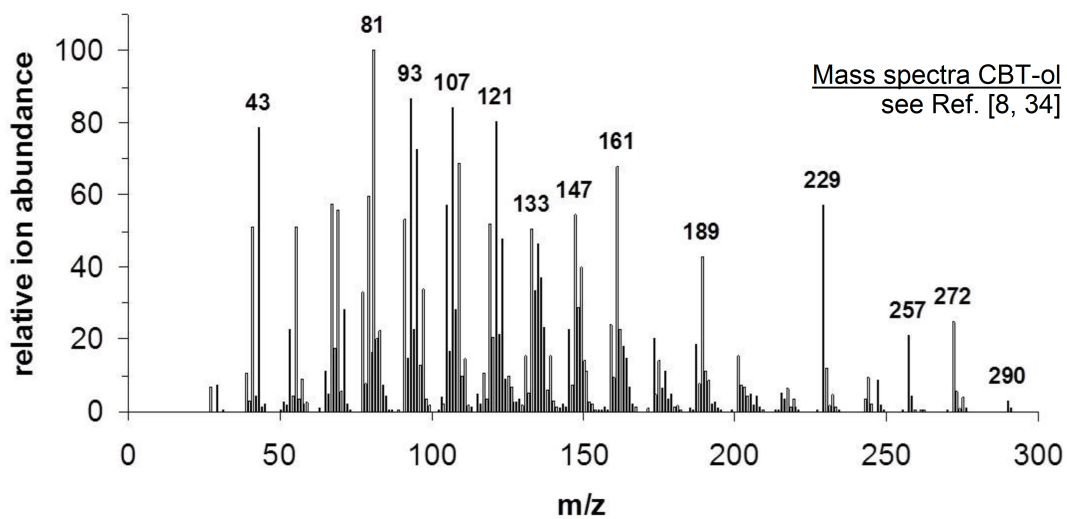**B**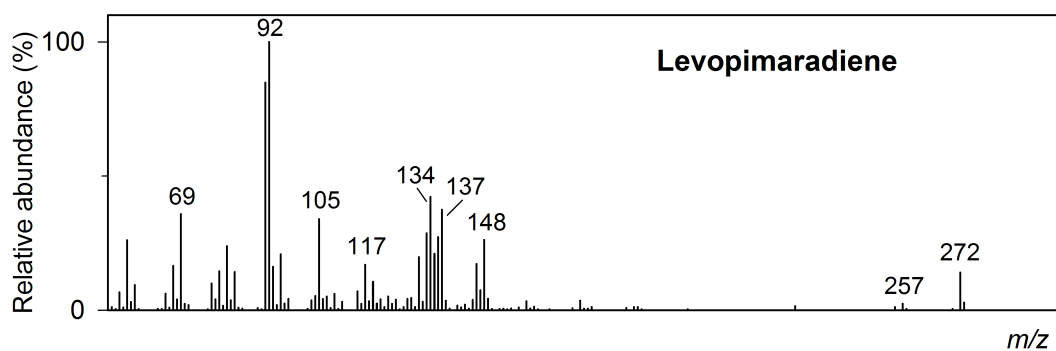**C**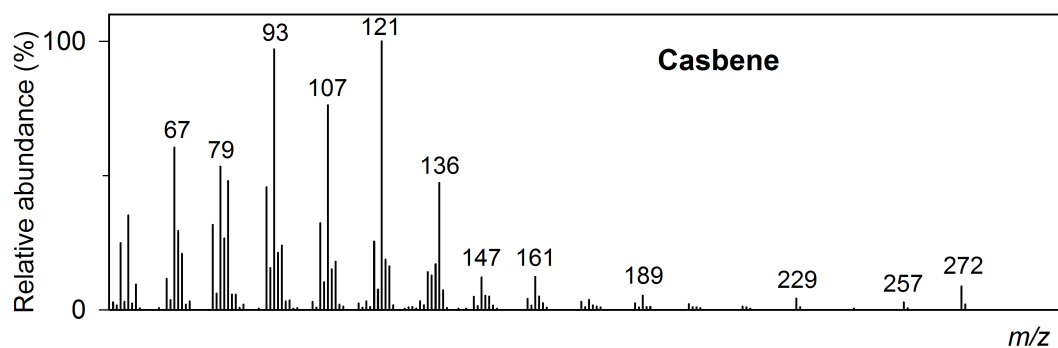

Supplement: Additional file 1 — Mass spectra recorded for the main products of the diterpene synthases investigated in this study: (A), CBT-ol, measured mass spectrum and published reference mass spectra; (B), Levopimaradiene; and (C) Casbene. [file 1746-4811-9-46-S1.pdf]

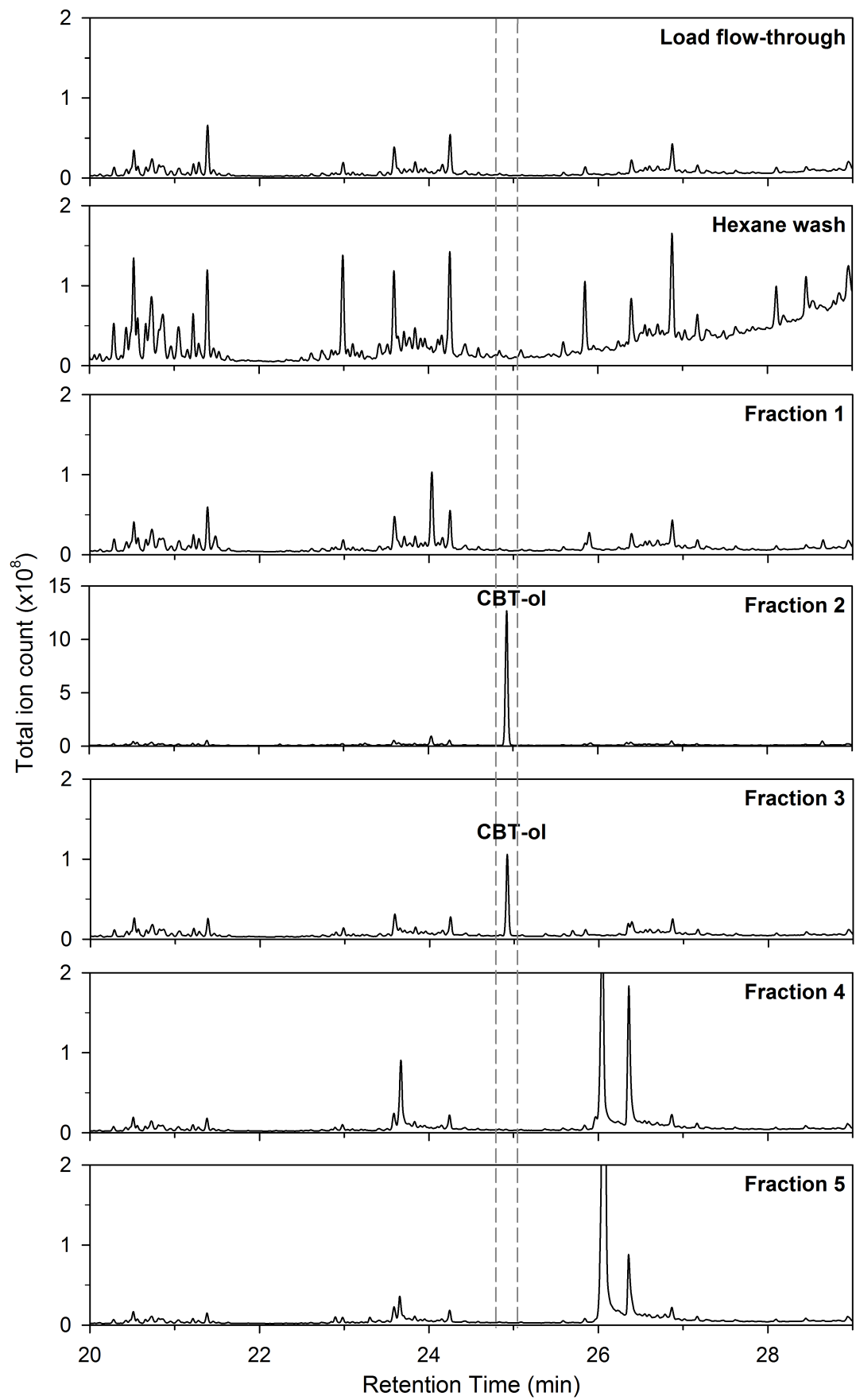

Supplement: Additional file 2 — GC-MS analysis of SPE eluates from total extracts of N. benthamiana leaves agro-infiltrated with p19 and NsCBTS2a . Treated leaves were harvested five days post-infiltration and crude extracts from six frozen leaf discs (9 mm in diameter) were purified over SPE column using hexane/ethyl-acetate/methanol (90:10:1, v/v) as eluent. The CBT-ol peak is framed by vertical dashed lines. [file 1746-4811-9-46-S2.pdf]

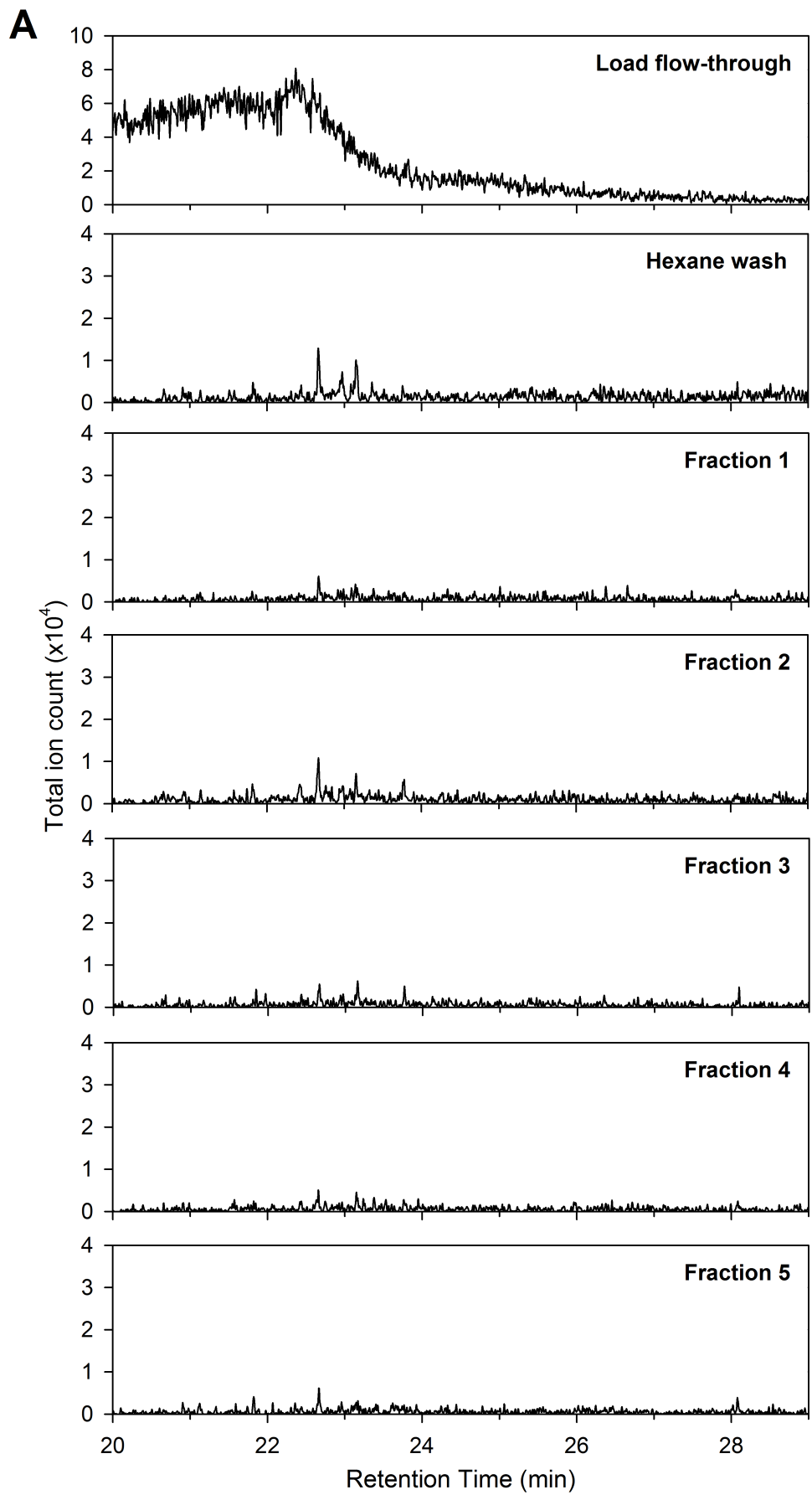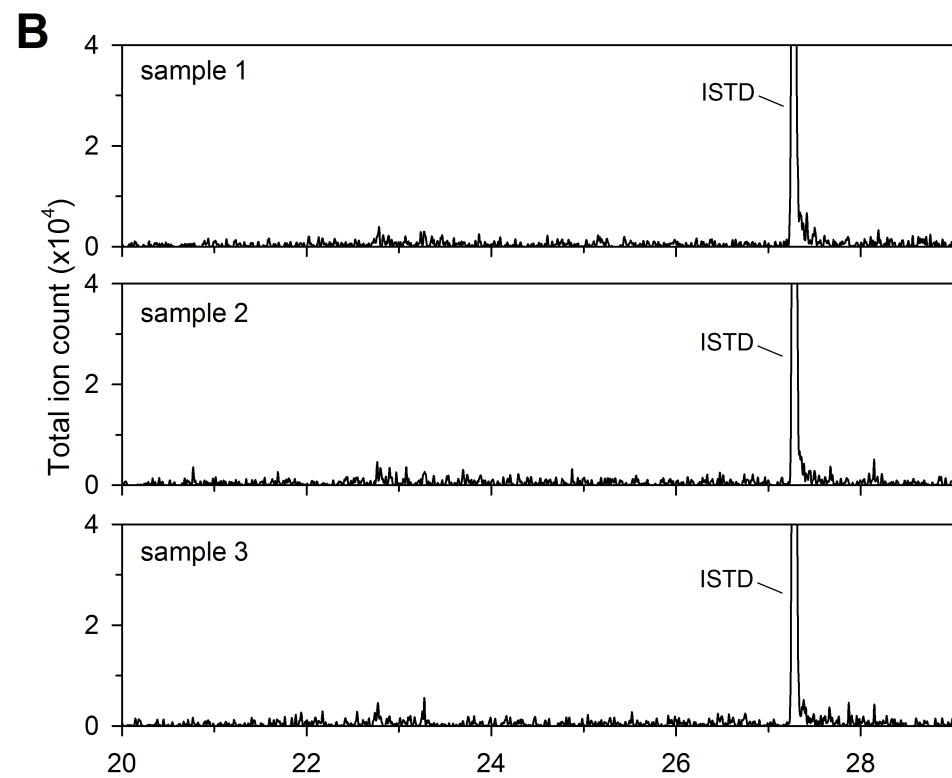

Supplement: Additional file 3 — GC-MS analysis of total extracts and hexane washes of N. benthamiana leaves co-expressing p19 and the empty T-DNA vector (pL1F-1) as a control. Treated leaves were harvested five days after infiltration of the plants. A) 272 m/z extracted spectra of SPE eluates from total leaf extracts after purification over SPE column. B) 272 m/z extracted total ion chromatograms of hexane washes from three independent leaf samples. No specific peak for 272 m/z was detected in the extracts indicating that no CBT-ol, casbene or levopimaradiene can be found in N. benthamiana upon co-expression of p19 and the empty T-DNA vector. [file 1746-4811-9-46-S3.pdf]

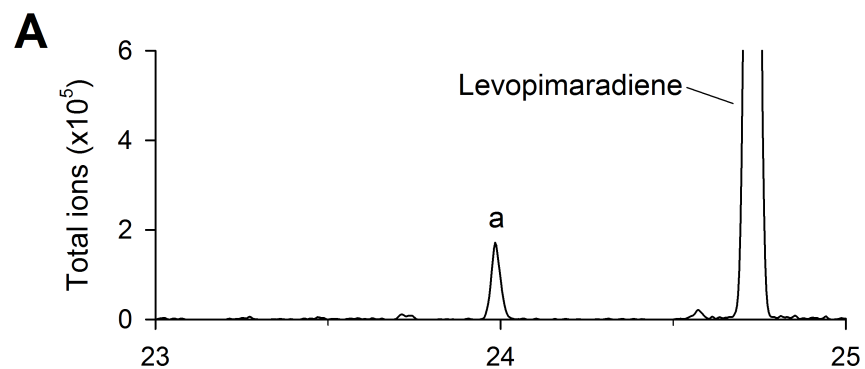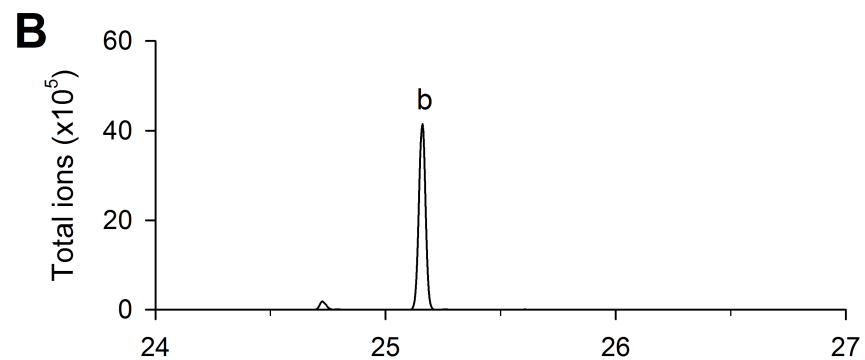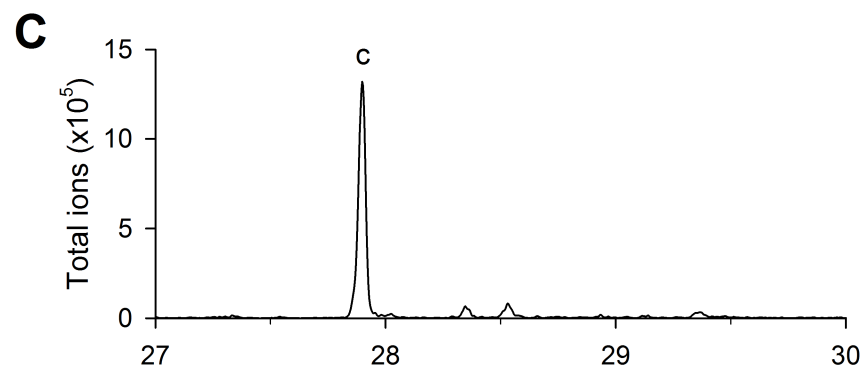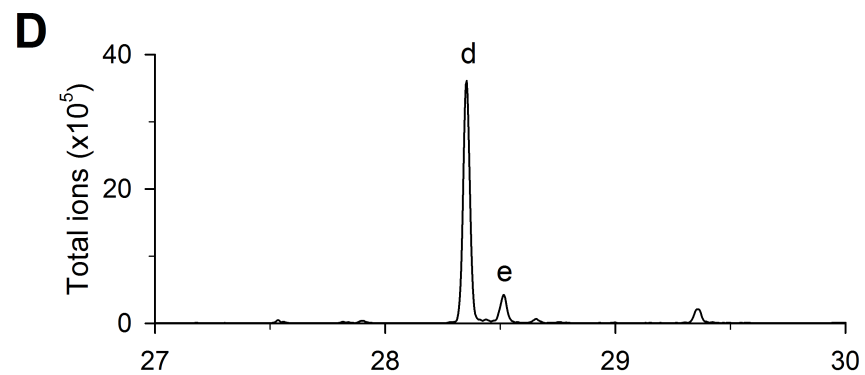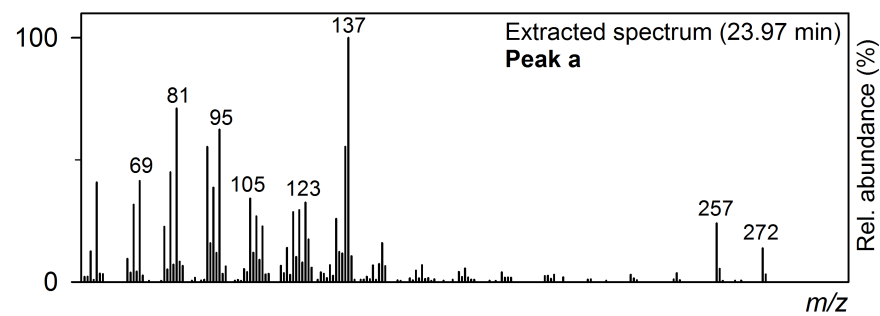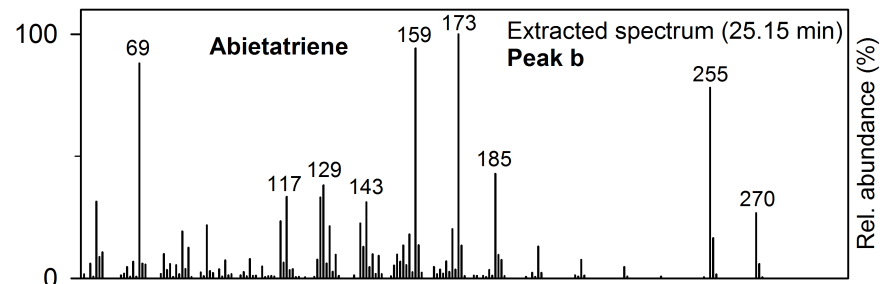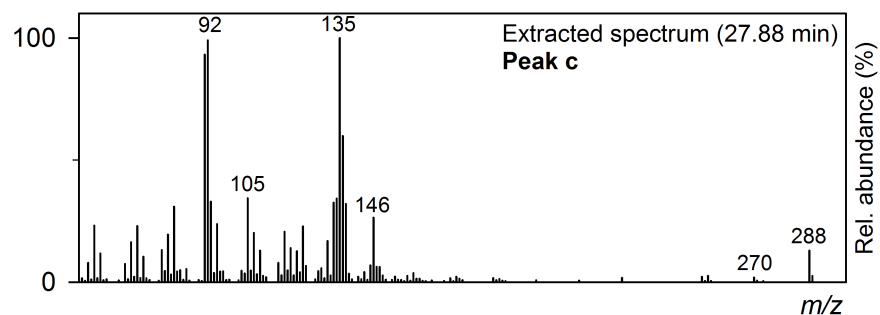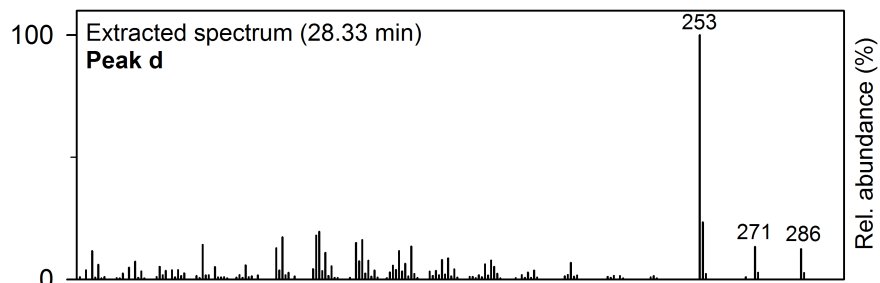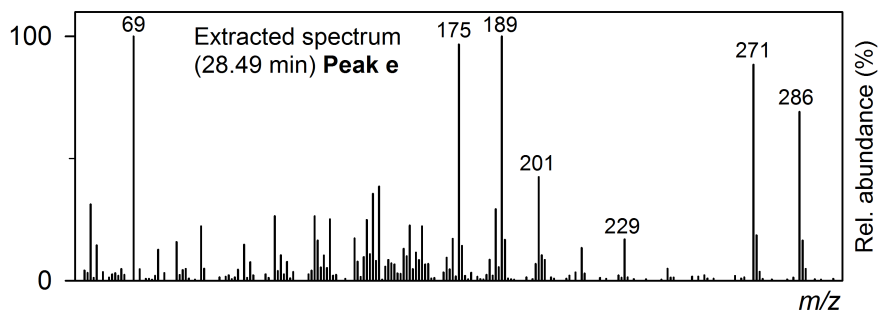

Supplement: Additional file 4 — GC-MS analysis of reaction products a – e which occurred in N. benthamiana leaves agro-infiltrated with p19 and GbLS . (A) 272 m/z extracted ion chromatograms and full scan mass spectra for diterpene-like compound peaking at 23.97 min (peak a). (B) 270 m/z extraction for peak b which was identified as Abietatriene using NIST MS search software v2.0 (http://chemdata.nist.gov/) (C) 288 m/z extracted chromatogram and corresponding mass spectrum for product peak c eluting after 27.88 min. (D) 286 m/z extracted ion chromatograms and the mass spectra recorded for compounds detected at 28.33 min (peak d) and 28.49 min (peak e), respectively. [file 1746-4811-9-46-S4.pdf]

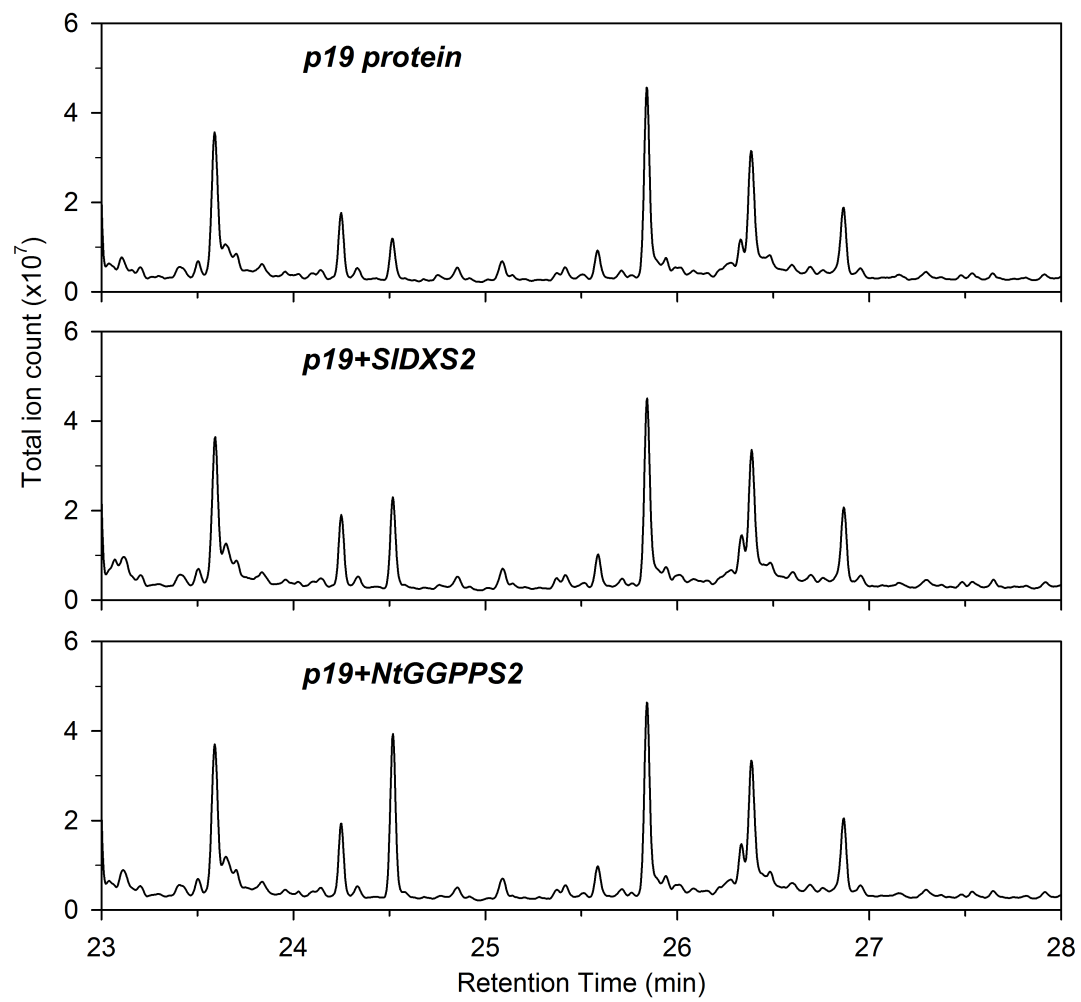

Supplement: Additional file 5 — Sections of total ion chromatograms obtained by GC-MS analysis of hexane washes from leaves infiltrated with p19 alone or co-infiltrated with p19 and SlDXS2 or NtGGPPS2 as controls. The infiltrated leaves were harvested five days post-infiltration. [file 1746-4811-9-46-S5.pdf]
